# Supplementary material for: Resting heart rate is a population-level biomarker of cardiorespiratory fitness: The Fenland Study
Source: PLoS One. 2023 May 11;18(5):e0285272. doi: 10.1371/journal.pone.0285272 (PMC10174582; doi:10.1371/journal.pone.0285272)
Supplement: S1 File — (DOCX) [file pone.0285272.s002.docx]

#

**SUPPLEMENTARY MATERIALS** (containing all supporting tables and figures for the manuscript)

**Resting heart rate is a biomarker of cardiorespiratory fitness: The Fenland Study.**

Tomas I. Gonzales^1^, Justin Y. Jeon^1,2^, Timothy Lindsay^1^, Kate Westgate^1^, Ignacio Perez-Pozuelo^1^ , Stefanie Hollidge^1^, Katrien Wijndaele^1^, Kirsten Rennie^1^, Nita Forouhi^1^, Simon Griffin^1^, Nick Wareham^1^, Soren Brage^1^

^1^ MRC Epidemiology Unit, University of Cambridge School of Clinical Medicine, Cambridge, UK

^2^ Department of Sport Industry Studies, Exercise Medicine Center for Diabetes and Cancer Patients (ICONS), Yonsei University, Seoul, Korea

# Supplementary Figure 1: Participant flow diagram. Cross-sectional analyses were performed on data from the first phase of the Fenland Study (Fenland I). Longitudinal analyses were performed on data from the second phase (Fenland II). Remote population monitoring of RHR was performed during the Fenland COVID-19 substudy.


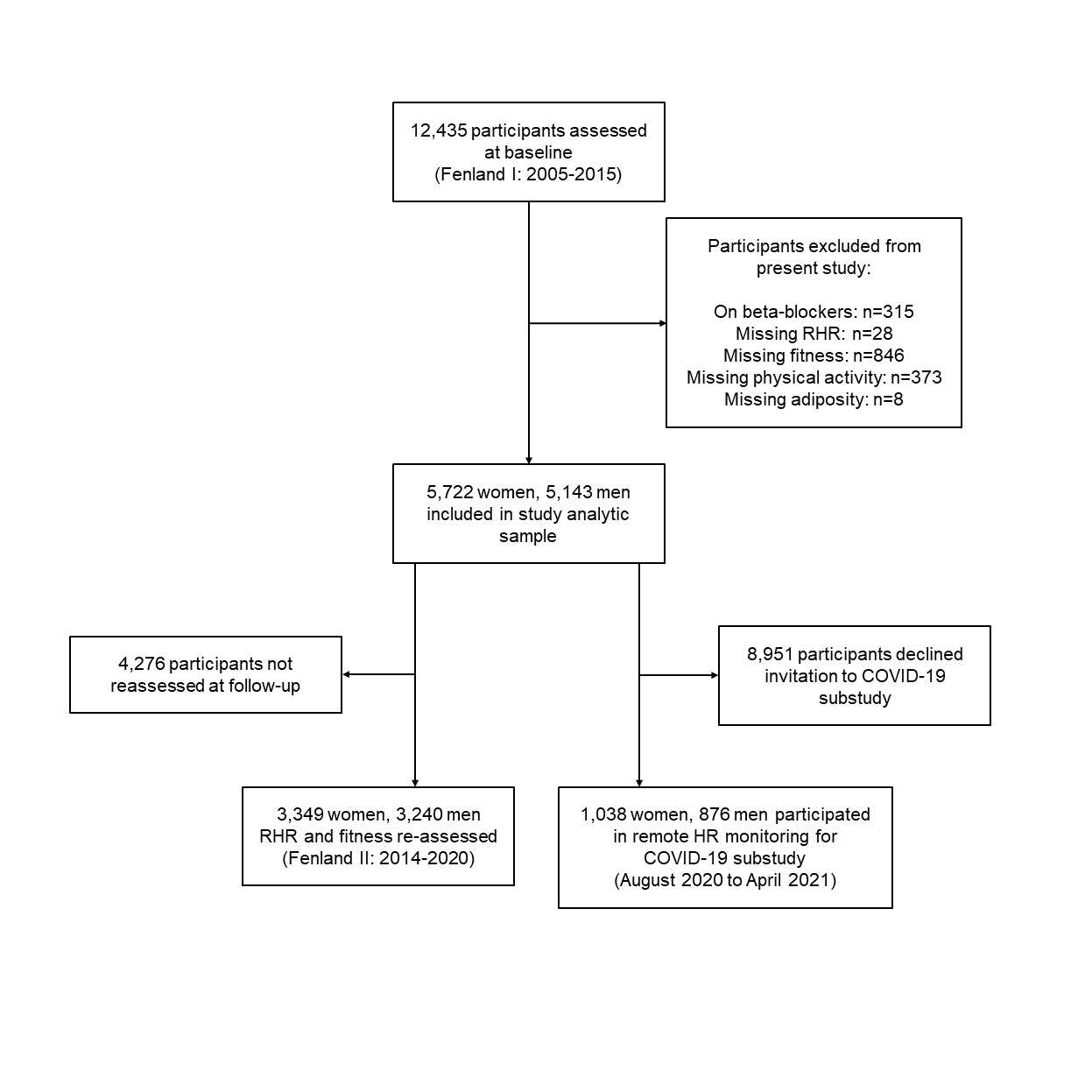


# Supplementary Figure 2: Associations between resting heart rate and maximal oxygen consumption expressed per kg fat-free mass, stratified by sex and adjusted for age (left column panels), age, ethnicity, smoking, alcohol consumption, and fat mass index (middle column panels), and further for physical activity energy expenditure (PAEE), moderate-vigorous PAEE (right column panels). Top: Sleeping resting heart rate. Middle: Supine resting heart rate. Bottom: Seated resting heart rate. The Fenland Study (n=10,865). Each point represents 5% of data in the binscatter plots.


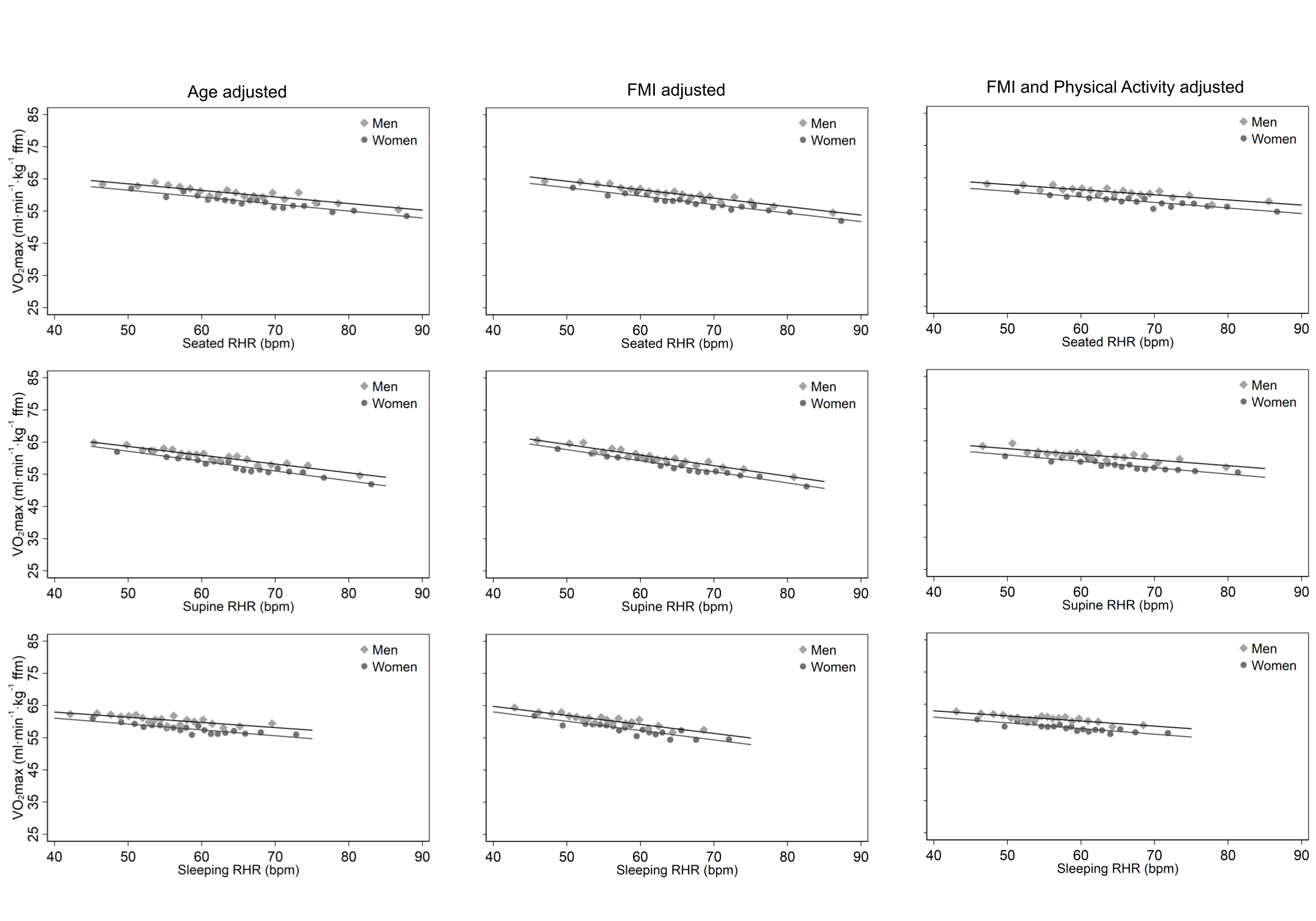


# Supplementary Figure 3: Linear spline regression modelling of RHR during the COVID-19 pandemic, stratified by sex and pre-pandemic RHR tertiles.


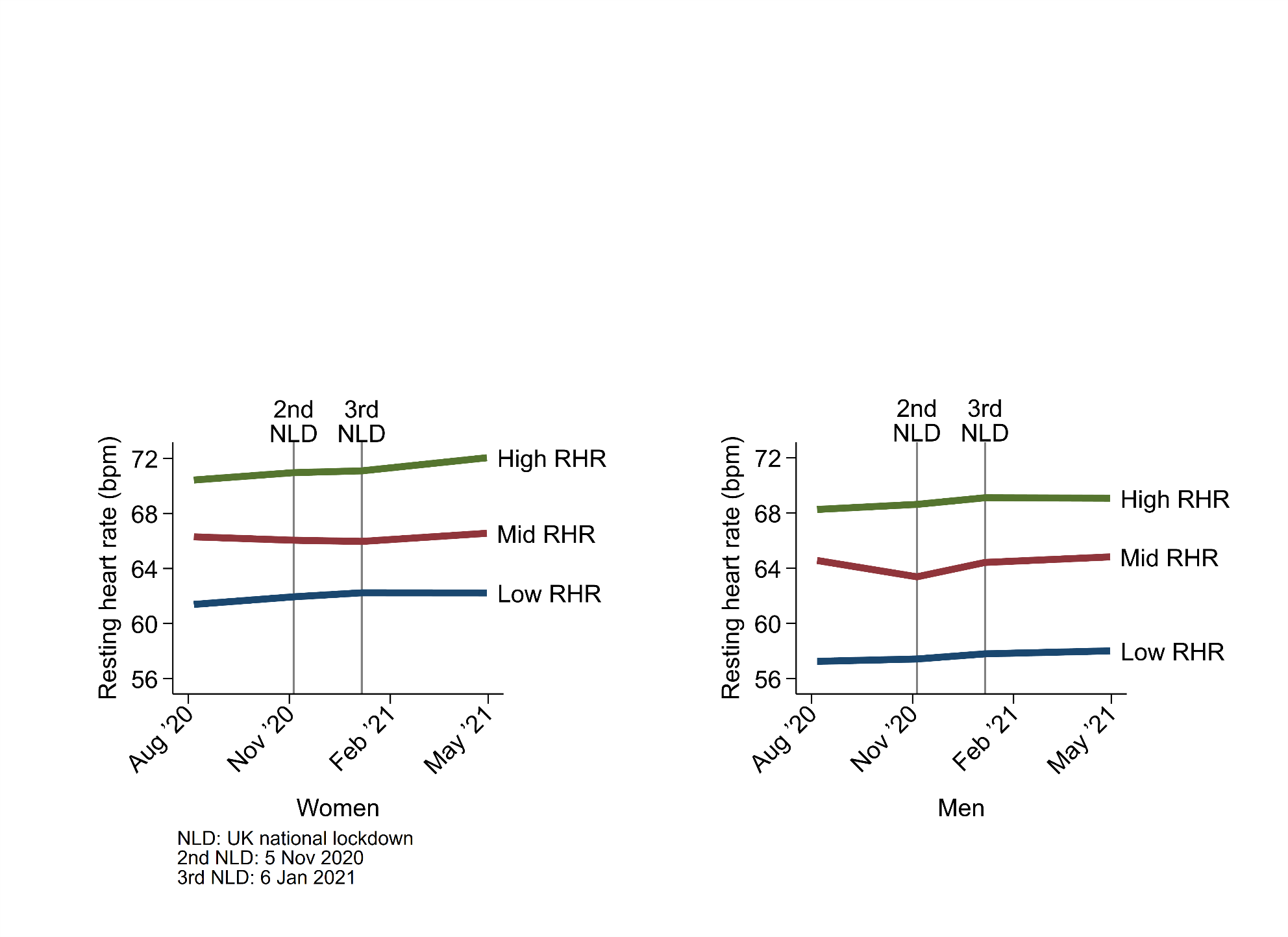


# Supplementary Figure 4: Linear spline regression modelling of RHR during the COVID-19 pandemic, stratified by sex and pre-pandemic BMI groups

#
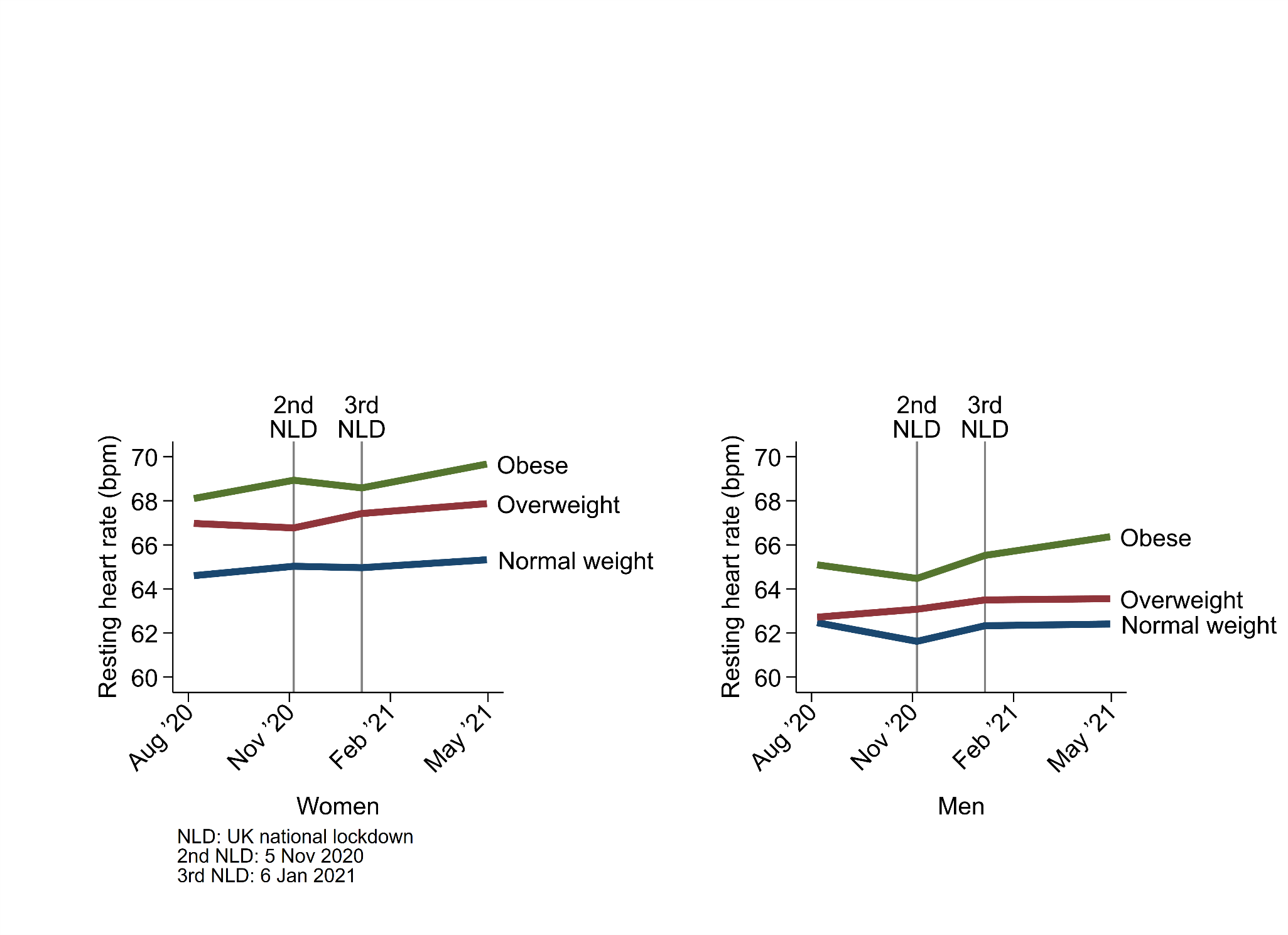
.

# Supplementary Table 1: Characteristics of complete cohort versus analytical sample in the Fenland study

|  | Fenland cohort (n=12,435) | Analytical sample (n=10,865) |
| --- | --- | --- |
| Demographics |  |  |
| Sex (W/M) | 6,691 / 5,744 | 5,772 / 5,143 |
| Age (years) | 48.6 (7.5) | 48.2 (7.4) |
| Ethnicity |  |  |
| White | 11,487 (92.4) | 10,062 (92.6) |
| Non-White | 948 (7.6) | 803 (7.4) |
| Smoker Status |  |  |
| Never smoked | 6,696 (53.9) | 5,894 (54.3) |
| Ex-smoker | 4,092 (32.9) | 3,568 (32.8) |
| Current smoker | 1,495 (12.0) | 1,280 (11.8) |
| Alcohol |  |  |
| <1/week | 4,196 (33.7) | 3,536 (32.5) |
| 1-4/week | 6,115 (49.2) | 5,472 (50.4) |
| Almost daily | 1,910 (15.4) | 1,686 (15.5) |
| RHR |  |  |
| Seated (bpm) | 66.3 (9.8) | 66.1 (9.4) |
| Supine (bpm) | 63.3 (9.0) | 63.1 (8.6) |
| Sleeping (bpm) | 57.1 (7.0) | 56.9 (6.9) |
| Anthropometrics |  |  |
| Height (m) | 1.70 (0.09) | 1.71 (0.09) |
| Body mass (kg) | 78.2 (16.3) | 77.7 (15.7) |
| BMI (kg/m^2^) | 26.9 (4.8) | 26.6 (4.5) |
| FMI (kg/m^2^) | 9.1 (3.9) | 8.9 (3.6) |
| Percent body fat (%) | 33.0 (9.2) | 32.5 (9.0) |
| Physical Activity |  |  |
| PAEE (kJ⋅day^-1^⋅kg^-1^) | 53.6 (22.1) | 54.6 (22.0) |
| MVPA (min⋅day^-1^) | 102.2 (78.7) | 105.0 (78.6) |
| MVPA (kJ⋅day^-1^⋅kg^-1^) | 21.4 (17.5) | 22.0 (17.6) |
| Beta-blocker | 315 (2.5) | 0 (0) |
|  |  |  |
| Values are means (standard deviation) or counts (%). RHR: Resting heart rate, bpm: beat per minute, BMI: Body mass index, FMI: Fat mass index, PAEE: Physical activity energy expenditure. MVPA: Moderate to vigorous physical activity. | | |

**Supplementary Table 2**: Baseline participant characteristics in women and men by age-adjusted and sex-specific cardiorespiratory fitness tertiles. The Fenland Study.

| Women (n = 5,143) | | | | |
| --- | --- | --- | --- | --- |
|  | Low fitness | Mid fitness | High fitness | Total |
|  | Mean ± SD | Mean ± SD | Mean ± SD | Mean ± SD |
| RHR |  |  |  |  |
| Seated (bpm) | 70.4 ± 9.1 | 67.0 ± 8.3 | 65.1 ± 8.7 | 67.5 ± 9.0 |
| Supine(bpm) | 67.5 ± 8.2 | 63.9 ± 7.6 | 62.0 ± 7.9 | 64.5 ± 8.2 |
| Sleeping (bpm) | 60.2 ± 6.5 | 58.1 ± 6.2 | 56.8 ± 6.7 | 58.4 ± 6.6 |
| Cardiorespiratory fitness |  |  |  |  |
| VO_2_max per kg BM | 27.8 ± 3.4 | 35.1 ± 1.9 | 45.5 ± 7.4 | 36.2 ± 8.7 |
| VO_2_max per kg FFM | 47.8 ± 7.1 | 56.2 ± 6.7 | 69.2 ± 12.7 | 57.7 ± 12.8 |
| Anthropometrics |  |  |  |  |
| Height (m) | 1.63 ± 0.06 | 1.64 ± 0.06 | 1.65 ± 0.06 | 1.64 ± 0.06 |
| Body mass (kg) | 75.6 ± 15.7 | 69.6 ± 12.3 | 66.0 ± 10.9 | 70.4 ± 13.7 |
| BMI (kg/m^2^) | 28.3 ± 5.6 | 25.8 ± 4.3 | 24.3 ± 3.8 | 26.1 ± 4.9 |
| FMI (kg/m^2^) | 12.0 ± 4.2 | 9.7 ± 3.4 | 8.4 ± 3.2 | 10.0 ± 3.9 |
| Percent body fat (%) | 41.1 ± 7.0 | 36.8 ± 7.2 | 33.5 ± 7.8 | 37.1 ± 8.0 |
| Physical Activity |  |  |  |  |
| PAEE kJ/day/kg | 41.8 ± 15.7 | 50.5 ± 17.4 | 59.3 ± 21.9 | 50.5 ± 19.8 |
| MVPA (min/day) | 53.2 ± 42.1 | 84.5 ± 53.7 | 122.6 ± 81.9 | 86.8 ± 67.8 |
| MVPA (kJ/day/kg) | 10.2 ± 8.6 | 16.9 ± 11.3 | 25.6 ± 18.0 | 17.6 ± 14.6 |
| Age (years) | 48.3 ± 7.4 | 48.2 ± 7.3 | 48.1 ± 7.3 | 48.2 ± 7.4 |
|  |  |  |  |  |
|  | Count (%) | Count (%) | Count (%) | Count (%) |
| Ethnicity |  |  |  |  |
| White | 1710(29.9) | 1774(31.0) | 1822(31.8) | 5306 (92.7) |
| Non-White | 199(3.5) | 133(2.3) | 84(1.5) | 416 (7.3) |
| Smoker Status |  |  |  |  |
| Never smoked | 1140(19.9) | 1063(18.6) | 1013(17.7) | 3216(56.2) |
| Ex-smoker | 596(10.4) | 631(11.0) | 603(10.5) | 1830(32.0) |
| Current smoker | 149(2.6) | 185(3.2) | 274(4.8) | 608(10.6) |
| Unknown | 24(0.4) | 28(0.5) | 16(0.3) | 68(1.2) |
| Alcohol Consumption |  |  |  |  |
| <1/week | 871(15.2) | 680(11.9) | 673(11.8) | 2224(38.9) |
| 1-4/week | 827(14.5) | 958(16.7) | 917(16.0) | 2702(47.2) |
| Almost daily | 179(3.1) | 230(4.0) | 286(5.0) | 695(12.1) |
| Unknown | 32(0.6) | 39(0.7) | 30(0.5) | 101(1.8) |
|  |  |  |  |  |
|  | | | | |

(**Supplementary Table 2:** Continued)

| Men (n = 5,722) | | | | |
| --- | --- | --- | --- | --- |
|  | Low fitness | Mid fitness | High fitness | Total |
|  | Mean ± SD | Mean ± SD | Mean ± SD | Mean ± SD |
| RHR |  |  |  |  |
| Seated (bpm) | 68.0 ± 9.9 | 63.8 ± 8.7 | 61.6 ± 9.1 | 64.5 ± 9.6 |
| Supine(bpm) | 65.0 ± 8.7 | 60.8 ± 7.9 | 58.7 ± 8.2 | 61.5 ± 8.7 |
| Sleeping (bpm) | 57.0 ± 6.5 | 54.7 ± 6.4 | 53.8 ± 6.8 | 55.2 ± 6.7 |
| Cardiorespiratory fitness |  |  |  |  |
| VO_2_max per kg BM | 34.8 ± 4.2 | 42.9 ± 2.3 | 54.0 ± 7.2 | 43.9 ± 9.3 |
| VO_2_max per kg FFM | 50.1 ± 6.8 | 59.4 ± 5.7 | 72.1 ± 11.1 | 60.5 ± 12.2 |
| Anthropometrics |  |  |  |  |
| Height (cm) | 1.77 ± 0.07 | 1.78 ± 0.07 | 1.79 ± 0.07 | 1.78 ± 0.07 |
| Body mass (kg) | 89.0 ± 15.8 | 85.5 ± 12.4 | 82.9 ± 11.9 | 85.8 ± 13.7 |
| BMI (kg/m^2^) | 28.3 ± 4.6 | 27.0 ± 3.5 | 26.2 ± 3.4 | 27.2 ± 4.0 |
| FMI (kg/m^2^) | 8.7 ± 3.0 | 7.5 ± 2.5 | 6.6 ± 2.5 | 7.6 ± 2.8 |
| Percent body fat (%) | 30.0 ± 6.5 | 27.2 ± 6.4 | 24.6 ± 7.0 | 27.3 ± 7.0 |
| Physical Activity |  |  |  |  |
| PAEE kJ/day/kg | 48.6 ± 18.5 | 59.8 ± 21.7 | 69.0 ± 24.8 | 59.1 ± 23.4 |
| MVPA (min/day) | 83.5 ± 55.4 | 126.0 ± 76.6 | 166.1 ± 95.5 | 125.2 ± 84.6 |
| MVPA (kJ/day/kg) | 17.2 ± 12.1 | 27.1 ± 17.0 | 36.7 ± 21.8 | 27.0 ± 19.1 |
| Age (years) | 48.4 ± 7.6 | 48.2 ± 7.6 | 48.1 ± 7.5 | 48.2 ± 7.5 |
|  |  |  |  |  |
|  | Count (%) | Count (%) | Count (%) | Count (%) |
| Ethnicity |  |  |  |  |
| White | 1,532(29.8) | 1,587(30.9) | 1,637(31.8) | 4,756(92.5) |
| Non-White | 183(3.6) | 127(2.5) | 77(1.5) | 387(7.5) |
| Smoker Status |  |  |  |  |
| Never smoked | 942(18.3) | 899(17.5) | 837(16.3) | 2,678(52.1) |
| Ex-smoker | 578(11.2) | 587(11.4) | 573(11.1) | 1,738(33.8) |
| Current smoker | 175(3.4) | 208(4.0) | 289(5.6) | 672(13.1) |
| Unknown | 20(0.4) | 20(0.4) | 15(0.3) | 55(1.1) |
| Alcohol Consumption |  |  |  |  |
| <1/week | 504(9.8) | 426(8.3) | 382(7.4) | 1,312(25.5) |
| 1-4/week | 899(17.5) | 945(18.4) | 926(18.0) | 2,770(53.9) |
| Almost daily | 287(5.6) | 325(6.3) | 379(7.4) | 991(19.3) |
| Unknown | 25(0.5) | 18(0.3) | 27(0.5) | 70(1.4) |
|  |  |  |  |  |
| SD: Standard deviation, RHR: Resting heart rate, bpm: beat per minute, BMI: Body mass index, FMI: Fat mass index, PAEE: Physical activity energy expenditure. VO_2_max: Estimated maximal oxygen consumption, MVPA: Moderate to vigorous physical activity, BM: Whole body mass, FFM: Fat free mass. Stratification by age-adjusted and sex-specific fitness tertiles. | | | | |

|  |  |  |  |  |
| --- | --- | --- | --- | --- |
|  |  |  |  |  |

# Supplementary Table 3: Regression equations documenting interrelationships between different measures of resting heart rate (RHR). These allow RHR measured in the seated or supine position to be related to RHR measured during sleep. Equations are pooled across sexes. The Fenland Study (n=10,865).

| Equation | r | RMSE (bpm) |
| --- | --- | --- |
| Supine RHR = 0.74 seated RHR + 14 | 0.81 | 5.1 |
| Supine RHR = 0.95 seated RHR | 0.81 | 5.4 |
| Sleeping HR = 0.58 supine RHR + 21 | 0.72 | 4.8 |
| Sleeping HR = 0.90 supine RHR | 0.72 | 5.5 |
| Sleeping HR = 0.47 seated RHR + 26 | 0.65 | 5.2 |
| Sleeping HR = 0.85 seated RHR | 0.65 | 6.3 |
| r: Pearson’s correlation coefficient RMSE: Root mean squared error | | |

# Supplementary Table 4: Association between resting heart rate (independent variable) and estimated maximal oxygen consumption expressed per kg fat-free mass (dependent variable). The Fenland Study.

|  |  | Seated RHR | | Supine RHR | | Sleeping RHR | |
| --- | --- | --- | --- | --- | --- | --- | --- |
|  |  | Men | Women | Men | Women | Men | Women |
|  | Total sample | | | | | | |
|  |  |  |  |  |  |  |  |
| Model 1 |  | -0.21 (-0.25, -0.18) | -0.22 (-0.25, -0.18) | -0.28 (-0.32, -0.24) | -0.30 (-0.34, -0.26) | -0.17 (-0.22, -0.12) | -0.18 (-0.23, -0.13) |
| Model 2 |  | -0.21 (-0.25, -0.18) | -0.21 (-0.25, -0.18) | -0.27 (-0.31, -0.23) | -0.29 (-0.33, -0.26) | -0.20 (-0.25, -0.15) | -0.21 (-0.26, -0.16) |
| Model 3 |  | -0.27 (-0.30, -0.23) | -0.26 (-0.30, -0.22) | -0.33 (-0.37, -0.29) | -0.34 (-0.38, -0.30) | -0.28 (-0.33, -0.22) | -0.29 (-0.34, -0.23) |
| Model 4 |  | -0.19 (-0.23, -0.16) | -0.19 (-0.22, -0.15) | -0.22 (-0.25, -0.18) | -0.23 (-0.27, -0.19) | -0.18 (-0.23, -0.13) | -0.19 (-0.24, -0.14) |
| Model 5 |  | -0.17 (-0.20, -0.13) | -0.17 (-0.21, -0.14) | -0.18 (-0.22, -0.14) | -0.20 (-0.24, -0.16) | -0.16 (-0.20, -0.11) | -0.18 (-0.22, -0.13) |
|  |  |  |  |  |  |  |  |
|  | Age stratified | | | | | | |
|  |  |  |  |  |  |  |  |
| Model 2 | <50 years | -0.19 (-0.24, -0.13) | -0.21 (-0.27, -0.15) | -0.25 (-0.31, -0.20) | -0.26 (-0.32, -0.20) | -0.20 (-0.27, -0.12) | -0.19 (-0.26, -0.11) |
|  | 50-60 years | -0.21 (-0.26, -0.16) | -0.21 (-0.26, -0.15) | -0.25 (-0.31, -0.19) | -0.31 (-0.37, -0.24) | -0.16 (-0.24, -0.08) | -0.21 (-0.29, -0.13) |
|  | >60 years | -0.26 (-0.33, -0.18) | -0.24 (-0.33, -0.16) | -0.32 (-0.41, -0.24) | -0.34 (-0.44, -0.25) | -0.29 (-0.40, -0.17) | -0.26 (-0.38, -0.14) |
|  |  |  |  |  |  |  |  |
| Model 3 | <50 years | -0.24 (-0.29, -0.18) | -0.24 (-0.30, -0.19) | -0.32 (-0.37, -0.26) | -0.30 (-0.36, -0.24) | -0.28 (-0.36, -0.20) | -0.24 (-0.32, -0.16) |
|  | 50-60 years | -0.28 (-0.33, -0.22) | -0.27 (-0.32, -0.21) | -0.32 (-0.38, -0.26) | -0.36 (-0.42, -0.30) | -0.24 (-0.33, -0.16) | -0.30 (-0.38, -0.22) |
|  | >60 years | -0.30 (-0.37, -0.22) | -0.29 (-0.38, -0.21) | -0.37 (-0.45, -0.28) | -0.39 (-0.49, -0.30) | -0.32 (-0.44, -0.21) | -0.36 (-0.48, -0.24) |
|  |  |  |  |  |  |  |  |
| Model 5 | <50 years | -0.13 (-0.18, -0.08) | -0.13 (-0.19, -0.08) | -0.15 (-0.21, -0.09) | -0.13 (-0.19, -0.07) | -0.13 (-0.21, -0.06) | -0.11 (-0.19, -0.04) |
|  | 50-60 years | -0.18 (-0.23, -0.13) | -0.17 (-0.22, -0.11) | -0.17 (-0.23, -0.11) | -0.21 (-0.27, -0.15) | -0.14 (-0.22, -0.06) | -0.17 (-0.25, -0.09) |
|  | >60 years | -0.20 (-0.27, -0.12) | -0.25 (-0.34, -0.17) | -0.22 (-0.31, -0.14) | -0.32 (-0.41, -0.22) | -0.21 (-0.32, -0.10) | -0.31 (-0.43, -0.19) |
|  |  |  |  |  |  |  |  |
|  | BMI stratified | | | | | | |
|  |  |  |  |  |  |  |  |
| Model 3 | <25 kg/m^2^ | -0.33 (-0.39, -0.27) | -0.29 (-0.34, -0.24) | -0.37 (-0.44, -0.31) | -0.38 (-0.44, -0.33) | -0.32 (-0.41, -0.23) | -0.36 (-0.43, -0.29) |
|  | 25-30 kg/m^2^ | -0.24 (-0.29, -0.19) | -0.27 (-0.34, -0.19) | -0.33 (-0.38, -0.27) | -0.32 (-0.39, -0.24) | -0.27 (-0.34, -0.19) | -0.23 (-0.32, -0.13) |
|  | >30 kg/m^2^ | -0.24 (-0.31, -0.16) | -0.19 (-0.28, -0.11) | -0.29 (-0.38, -0.21) | -0.31 (-0.39, -0.22) | -0.24 (-0.35, -0.12) | -0.21 (-0.33, -0.10) |
|  |  |  |  |  |  |  |  |
| Model 5 | <25 kg/m^2^ | -0.20 (-0.26, -0.14) | -0.17 (-0.22, -0.12) | -0.19 (-0.26, -0.12) | -0.21 (-0.27, -0.16) | -0.17 (-0.25, -0.08) | -0.23 (-0.30, -0.16) |
|  | 25-30 kg/m^2^ | -0.15 (-0.20, -0.11) | -0.19 (-0.26, -0.12) | -0.19 (-0.25, -0.14) | -0.20 (-0.27, -0.12) | -0.18 (-0.25, -0.11) | -0.13 (-0.22, -0.04) |
|  | >30 kg/m^2^ | -0.14 (-0.21, -0.06) | -0.14 (-0.22, -0.06) | -0.16 (-0.24, -0.07) | -0.21 (-0.30, -0.12) | -0.11 (-0.22, -0.00) | -0.14 (-0.25, -0.03) |
|  |  |  |  |  |  |  |  |
|  | PAEE stratified | | | | | | |
|  |  |  |  |  |  |  |  |
| Model 5 | <40 kJ/day/kg | -0.16 (-0.23, -0.10) | -0.24 (-0.30, -0.18) | -0.23 (-0.31, -0.15) | -0.30 (-0.36, -0.23) | -0.13 (-0.24, -0.03) | -0.20 (-0.29, -0.11) |
|  | 40-60 kJ/day/kg | -0.20 (-0.25, -0.14) | -0.15 (-0.20, -0.09) | -0.22 (-0.28, -0.15) | -0.17 (-0.23, -0.11) | -0.17 (-0.25, -0.09) | -0.17 (-0.25, -0.10) |
|  | >60 kJ/day/kg | -0.13 (-0.19, -0.08) | -0.10 (-0.17, -0.03) | -0.11 (-0.17, -0.05) | -0.11 (-0.18, -0.03) | -0.16 (-0.23, -0.09) | -0.16 (-0.25, -0.07) |
|  |  |  |  |  |  |  |  |

Reported values are beta coefficients (95% confidence interval) for the difference in fitness (dependent variable) per a 1-bpm difference in resting heart rate (independent variable).

Model 1: Age adjusted

Model 2: Model 1 + ethnicity, smoking and alcohol adjusted

Model 3: Model 2 + fat mass index adjusted

Model 4: Model 3 + physical activity energy expenditure (PAEE) adjusted

Model 5: Model 4 + moderate-vigorous intensity PAEE adjusted

# Supplementary Table 5: Association between resting heart rate and directly measured maximal oxygen consumption expressed per kg total-body mass. Analysis was conducted in a sub-sample of participants (43 women; 42 men) with direct measurements of VO_2_max, assessed using a maximal treadmill test and measured using a metabolic cart (Jaeger Oxycon Pro).

|  | Seated RHR | | Supine RHR | | Sleeping RHR | |
| --- | --- | --- | --- | --- | --- | --- |
|  | Men | Women | Men | Women | Men | Women |
| Model A | -0.19(-0.38, 0.0031) | -0.25(-0.44, -0.072) | -0.19(-0.42, 0.030) | -0.32(-0.51, -0.13) | -0.26(-0.57, 0.42) | -0.42(-0.64, -0.20) |
| Model B | -0.23(-0.39, -0.068) | -0.23(-0.42, -0.050) | -0.23(-0.41, -0.039) | -0.29(-0.49, -0.094) | -0.33(-0.58, -0.078) | -0.40(-0.62, -0.18) |
|  |  |  |  |  |  |  |
| Reported values are beta coefficients (95%CI) for the difference in estimated fitness (dependent variable) per a 1-bpm difference in resting heart rate (independent variable).  Model A: Age adjusted  Model B: Age and BMI adjusted For reference see: Gonzales TI, Westgate K, Hollidge S, Lindsay T, Jeon J, Brage S. Estimating maximal oxygen consumption from heart rate response to submaximal ramped treadmill test. medRxiv 2020; 2020.02.18.20024489. | | | | | | |
