# Supplementary material for: Resting heart rate is a population-level biomarker of cardiorespiratory fitness: The Fenland Study
Source: PLoS One. 2023 May 11;18(5):e0285272. doi: 10.1371/journal.pone.0285272 (PMC10174582; doi:10.1371/journal.pone.0285272)
Supplement: Attachment — Submitted filename: 3. First_Rebuttal_Letter.docx [file pone.0285272.s003.docx]

Dear Editor,

We thank the Reviewers for their comments on our manuscript. We have responded to each of these in turn and revised our manuscript where appropriate. We hope that our work will be of interest to the readers of the Journal.

Sincerely Yours,

Soren Brage

**Reviewer #1 Comments**

This study has a challenge to provide the information that RHR is a valid population-level biomarker of cardiorespiratory fitness. As mentioned by the authors, the measurement of fitness is hard to conduct routinely in public health settings and daily lives. Given these backgrounds, the finding of this study may contribute to the relevant area. However, the reviewer could not catch the motivation of this study, and the consistency of manuscript is not sufficient. Therefore, it seems to be difficult for future readers to follow your study. You can address some comments as follows.

**Major comments**

1. The authors pointed out that the methodological reasons may result in a limited use of RHR as a population-level biomarker of fitness. Given this, how are the results of this study useful to address this problem? Throughout the study, there is no hypothesis or authors’ expectation. First, this study measured RHR while seated, supine, and during sleep. What is the merit to compare these three ways? What results did you expect? Given that seated and supine RHR were measured under an overnight fast condition, which is not a daily life, sleeping heart rate may be suitable for daily use. What does this comparison resolve for methodological problems?

RESPONSE:

We investigated the relationship between fitness and sleeping HR, supine RHR, and seated RHR in order to demonstrate that the fitness-to-RHR relationship will vary depending on how RHR is measured. We expected the relationship to differ because supine RHR is generally greater than seated RHR, which is greater than sleeping HR; we have quantified these differences here. These findings are worthwhile because they resolve the methodological problem of how to relate fitness to RHR measured in different ways. This methodological issue is problematic in population-based clinical research settings – where RHR could be measured differently across research centres and patient subgroups – however our findings ameliorate this issue.

RHR does not differ between the fed and fasted state (see https://doi.org/10.1016/j.ijcha.2019.100378). We agree that sleeping heart rate is very suitable for daily use and we also demonstrate the feasibility and validity of less standardised awake resting heart rate monitoring using smartphones which shows clear separation of the three fitness groups.

Second, what is the merit to examine the influence of modifiable factors on the RHR-to-fitness association? Please provide a reason why quantifying the influence on the RHR-to-fitness relationship strengthen the argument for using RHR as a population-level biomarker of fitness. There is a possibility that the criterion-related validity of RHR to fitness may improve when a factor affecting the association between RHR and fitness is considered. The relevant discussion (L301, 389-390) should be also modified with this viewpoint.

RESPONSE:

We investigated the influence of health factors on the fitness-to-RHR relationship in order to explore what may explain the association in populations with diverse health behaviours. We show in Table 2 that obesity attenuates the fitness-to-RHR relationship by approximately 14% and that physical activity additionally attenuates the relationship by approximately 33%. We also show that the relationship persists in different population strata including age, sex, obesity and physical activity. This information strengthens the use of RHR as a population-level biomarker of fitness in a diverse range of population subgroups.

Third, it is very hard to catch the role of data during the pandemic in this study (Figure 3 and Supplementary Figure 2 and 3). The relevant discussion (L294-299) should be also modified with this viewpoint. If necessary, it is better to delete this part in this paper.

RESPONSE:

The smartphone substudy demonstrates firstly that obtaining a measure of resting heart rate outside of laboratory settings using increasingly commonly available technology is feasible at scale. Secondly, the clear separation of smartphone-measured resting heart rate by baseline fitness supports the validity of this measurement as a biomarker of fitness. Thirdly, through serial measurements of resting heart rate during different phases of the pandemic, we were able to describe the different trajectories of the biomarker which reflect different opportunities for people to maintain or improve their fitness during the public health management of the Covid pandemic.

2. Related to the above-mentioned comment, the information of previous study in this area was limited in Introduction. Therefore, it is hard for future readers to follow the gap between this work and previous works. Why did not the authors cite the literatures (ref. 23, 24, 25) in Introduction? What is the difference between these studies and this study? Please explain.

RESPONSE:

These previous studies were focused on studying the relationship between fitness or RHR and various disease outcomes, but do not explore how the relationship between RHR and fitness is influenced by modifiable behaviours (i.e. adiposity, physical activity). Our study focuses on this latter question, which no previous large-scale study has addressed systematically. We now more clearly communicate this in the introduction.

3. For the main results in this study, all of data including fitness were assessed based on heart rate. This may lead to overestimate the association between RHR and fitness. Please further discuss this point.

RESPONSE:

We agree with this point and it was therefore the first point mentioned in the limitations section. However, we have validated our criterion measure of fitness against directly measured oxygen consumption during a maximal test, and in Supplementary Table 5 we provide additional results for the association between RHR and directly measure maximal oxygen consumption in the validation study. The values of the association are comparable to those attained in the main study, where fitness was estimated from exercise heart rate response to a submaximal exercise test.

**Minor comments**

Abstract

It is better to provide that the population during the coronavirus disease-2019 pandemic is also a sub-group of the UK Fenland Study.

We now document that the Covid study is a subsample of the Fenland study.

L39-40

Where are these results from? Please specify. (If these results are based on the cross-sectional analysis, why sex-adjusted?)

We thank the reviewer for identifying this reporting error. These lines now document the results from the first column of Figure 1.

L45-46

Is this a conclusion based on your findings? It is better to refer to your results.

We have now deleted this sentence.

Introduction

L73-75

Please provide a reason why RHR could a viable alternative. Is RHR correlated with VO2max? How much? Is RHR also associated with the lower risk of diabetes, CVD, cancer, and mortality?

We now provide a reason at the indicated location.

Methods

L102-103

Participants with diabetes were excluded. Why did the study exclude only diabetes? How about other chronic diseases such as hypertension and dyslipidemia? Moreover, what does terminal illness include?

The Fenland Study is a population-based cohort study designed to investigate the relationship between environmental and genetic factors on obesity, type 2 diabetes, and related metabolic disorders. Thus, participants with known prevalent type 2 diabetes were not invited since a main study aim is to examine the determinants of diabetes incidence. Other chronic conditions were not part of the exclusion criteria but if we would have excluded people with hypertension, for example, the recruited sample would have been substantially smaller and biased towards the healthy. As we recruited our sample through general practices, we asked the family doctor to exclude potential participants in their practice with a terminal illness that would prevent the patient from attending our clinical assessment; thus this was left to the clinical judgment of the family doctor.

Supplementary Table 1

It is better to provide each effect size instead of P value, because the number of participants is large.

We have decided to omit this column from Supplementary Table 1 since it uses mixed methods to compute P-values and may be difficult to interpret. If desired - readers may calculate P-values and effect sizes using the counts, means, and standard deviations that are provided. Note that the effect sizes for differences between samples are very low across most comparisons.

L109-L124

It is better to provide the flowchart of selection of participants, because the study was conducted in the same study population (the UK Fenland Study). The STROBE statement recommends the use of a flow diagram.

We now more thoroughly document exclusions in this section and provide a flow chart in a supplementary figure.

L134-138

Please provide the details (company, model number, etc.) of sensor measuring sleep heart rate.

The sensor used during sleep was the same combined heart rate and movement sensor used during free living and during the clinical visit (Actiheart, CamNtech, Papworth, UK); we have made this clearer at the indicated location.

L214-216d

Why did the authors exclude the missing continuous variables? It is better to use multiple imputation method.

This approach is not appropriate when imputing values for the main exposures and outcomes of a study (i.e. RHR, fitness). Therefore, we did not use multiple imputation for our main analyses. We could have used multiple imputation for missing adiposity and physical activity covariates but as the number of missing values were very low for these variables and our sample was very large, we chose to just conduct a complete-case analysis which has the added benefit that our stratified analyses of these groups have the same total sample size.

Results

L232-233

Is RHR is supine position? Please specify.

Here we are referring to RHR measured in general, not in a specific measurement modality; this is now clarified.

Supplementary Table 3

This table is very confusing. Do the results represent men and women? Why are regression equations needed? Isn't a correlation matrix sufficient?

This table documents interrelationships between different measures of RHR. Regression equations are needed to convert from one measure to another. The equations are not sex stratified. We now make this clear in the table header. The equations allow quantitative mapping between the different measures of resting heart rate.

Figure 1

Is “R” in the figure caption equal to “r” in each figure? Please match.

The figure caption has been edited.

**Reviewer #2 Comments**

The authors provide useful information for RHR as a biomarker. Particularly they provide findings on seated, supine, and sleeping heart rate.

These findings support the use of non-exercise fitness equations eCRF that use RHR as a key algorithm parameter. See, Wang Y, Chen S, Lavie CJ, Zhang J, Sui X. An overview of non-exercise estimated cardiorespiratory fitness: estimation equations, cross-validation and application. J Sci Sport Exerc 2019 Jun 10;1(1):38-53.

There are studies that provide correlations information between MAX VO2 and RHR. See Sloan R, Visentini-Scarzanella M, Sawada S, Sui X, Myers J, Estimating Cardiorespiratory Fitness Without Exercise Testing or Physical Activity Status in Healthy Adults: Regression Model Development and Validation, JMIR Public Health Surveill 2022;8(7):e34717

Artero et al 2014 showed that eCRF was a better predictor than of all cause mortality than RHR alone. RHR is more useful when applied to eCRF equations.

We thank the reviewer for their comments and for highlighting previous research related to the present study. We have incorporated these previous studies into the discussion where relevant.
