# Supplementary material for: Resting heart rate is a population-level biomarker of cardiorespiratory fitness: The Fenland Study
Source: PLoS One. 2023 May 11;18(5):e0285272. doi: 10.1371/journal.pone.0285272 (PMC10174582; doi:10.1371/journal.pone.0285272)
Supplement: Attachment — Submitted filename: 5. Second_Revision_rebuttal.docx [file pone.0285272.s004.docx]

Rebuttal, second revision

**Comments to the Author**

Reviewer #1: (No Response)

Reviewer #2: All comments have been addressed

Reviewer #3: (No Response)

2. Is the manuscript technically sound, and do the data support the conclusions?

Reviewer #1: Partly

Reviewer #2: Yes

Reviewer #3: Yes

3. Has the statistical analysis been performed appropriately and rigorously?

Reviewer #1: Yes

Reviewer #2: Yes

Reviewer #3: Yes

4. Have the authors made all data underlying the findings in their manuscript fully available?

Reviewer #1: Yes

Reviewer #2: Yes

Reviewer #3: Yes

5. Is the manuscript presented in an intelligible fashion and written in standard English?

Reviewer #1: Yes

Reviewer #2: Yes

Reviewer #3: Yes

6. Review Comments to the Author

Reviewer #1: (No Response)

Reviewer #2: I have no further comments to be made.

Reviewer #3: The reviewers comments have been addressed well.

Minor Comment:

Methods
The methods are clearly and thoroughly explained. However, in lines 164-165, I would like to see more detail regarding the treadmill protocol that was used. Currently, the protocol is very general in its description. Was this a standard treadmill protocol, i.e. Bruce test?

**RESPONSE**

We now provide additional text (in red) in the present manuscript to detail the treadmill protocol used – this information was available in the cited reference but we agree that it is more reader friendly to include the extra detail here. We now cite the previous work earlier in this paragraph and we have also provided an extra citation to a recently published article that described the validity and epidemiological utility of the fitness estimates arising from this protocol.
